# Competition for clinical cases in Alzheimer’s disease (CCCAD) – Janssen-Cilag

**DOI:** 10.1590/S1980-57642009DN20100015

**Published:** 2008

Janssen-Cilag, with the support of the Brazilian Academy of Neurology – ABN, the Brazilian Association of Psychiatry – ABP and the Brazilian Society of Geriatrics and Gerontology – SBGG, ran the Competition for Clinical Cases in Alzheimer’s Disease (CCCDA) throughout 2007.

The CCCAD was devised to foster and recognize the sharing of experience amongst medical professionals attending patients with Alzheimer’s Disease through descriptions of clinical cases with academic and/or scientific relevance.

This edition carries, in full, the case entitled “Dysexecutive mild cognitive impairment associated with frontal atrophy”, by Marcio Luiz Figueredo Balthazar, Benito Pereira Damasceno, which was winner of the CCCAD. The Abstract published below received honorable mention and followed the rules for publication in this journal.

## Treatment of mild cognitive impairment with galantamine

***Jerusa Smid, Ricardo Nitrini***

***Universidade de São Paulo***

### Abstract:

Mild cognitive impairment (MCI) is an intermediate stage between normal cognitive status and dementia. Treatment of MCI with cholinesterase inhibitors (ChEIs) or other medications remains a controversial issue, although many experts agree that ChEIs are effective in the treatment of MCI, especially the amnestic type. We report a case of a 74-year-old Brazilian man, with memory complaints and mild depressive symptoms, which were confirmed by family members but not severe enough to interfere with his daily life activities as a lawyer. Neuropsychological examination confirmed the memory impairment but also revealed initiation/perseveration deficit. He was treated with galantamine and sertraline (50 mg/day). When the maximum dose of galantamine (24 mg/day) was reached, improvement was observed by the patient, family members and medical staff. The patient had excellent evolution associated with this treatment and since then annually repeated neuropsychological evaluations have revealed memory impairment but no further cognitive decline over the last six years. For a short period of time, the doses of galantamine had to be reduced because the drug was not available for purchase. The patient and family members reported a clear cognitive decline during this period, which remitted when the habitual doses of 24 mg/day were reintroduced. Although it is not possible to be sure of the effect of galantamine or of the association of galantamine and sertraline on the evolution of this case, it is remarkable that his condition remained unchanged on comprehensive neuropsychological evaluations performed over a long period of time.

**Key words:** mild cognitive impairment, galantamine, Alzheimer’s disease

## Primary progressive aphasia: first manifestation of Alzheimer’s disease?

***João Francisco Villanova Fiqueiredo***

***Santa Casa de Misericórdia de São Paulo***

### Abstract:

A 73 year-old woman presented with progressive word-finding difficulty that had been occurring for over one year. Although she came to us as a primary progressive aphasia case, further investigation with a specific neuropsychological evaluation for language deficit patients revealed memory, praxis and executive function impairment. This case shows that progressive aphasia may be the first manifestation of Alzheimer's disease.

**Key words:** primary progressive aphasia, Alzheimer`s disease, language.

